# Utilization of Host Cell Chromosome Conformation by Viral Pathogens: Knowing When to Hold and When to Fold

**DOI:** 10.3389/fimmu.2021.633762

**Published:** 2021-03-25

**Authors:** Kinjal Majumder, Abigail J. Morales

**Affiliations:** ^1^ Institute for Molecular Virology and McArdle Laboratory for Cancer Research, Human Cancer Virology Program, University of Wisconsin Carbone Cancer Center, University of Wisconsin School of Medicine and Public Health, Madison, WI, United States; ^2^ Department of Medical Laboratory Sciences, Hunter College of the City University of New York, New York, NY, United States

**Keywords:** epigenetics, topologically associating domains, CTCF, Cohesin, B cells, human gammaherpesviruses, small DNA viruses

## Abstract

Though viruses have their own genomes, many depend on the nuclear environment of their hosts for replication and survival. A substantial body of work has therefore been devoted to understanding how viral and eukaryotic genomes interact. Recent advances in chromosome conformation capture technologies have provided unprecedented opportunities to visualize how mammalian genomes are organized and, by extension, how packaging of nuclear DNA impacts cellular processes. Recent studies have indicated that some viruses, upon entry into host cell nuclei, produce factors that alter host chromatin topology, and thus, impact the 3D organization of the host genome. Additionally, a variety of distinct viruses utilize host genome architectural factors to advance various aspects of their life cycles. Indeed, human gammaherpesviruses, known for establishing long-term reservoirs of latent infection in B lymphocytes, utilize 3D principles of genome folding to package their DNA and establish latency in host cells. This manipulation of host epigenetic machinery by latent viral genomes is etiologically linked to the onset of B cell oncogenesis. Small DNA viruses, by contrast, are tethered to distinct cellular sites that support virus expression and replication. Here, we briefly review the recent findings on how viruses and host genomes spatially communicate, and how this impacts virus-induced pathology.

## Introduction

**Figure 1:**
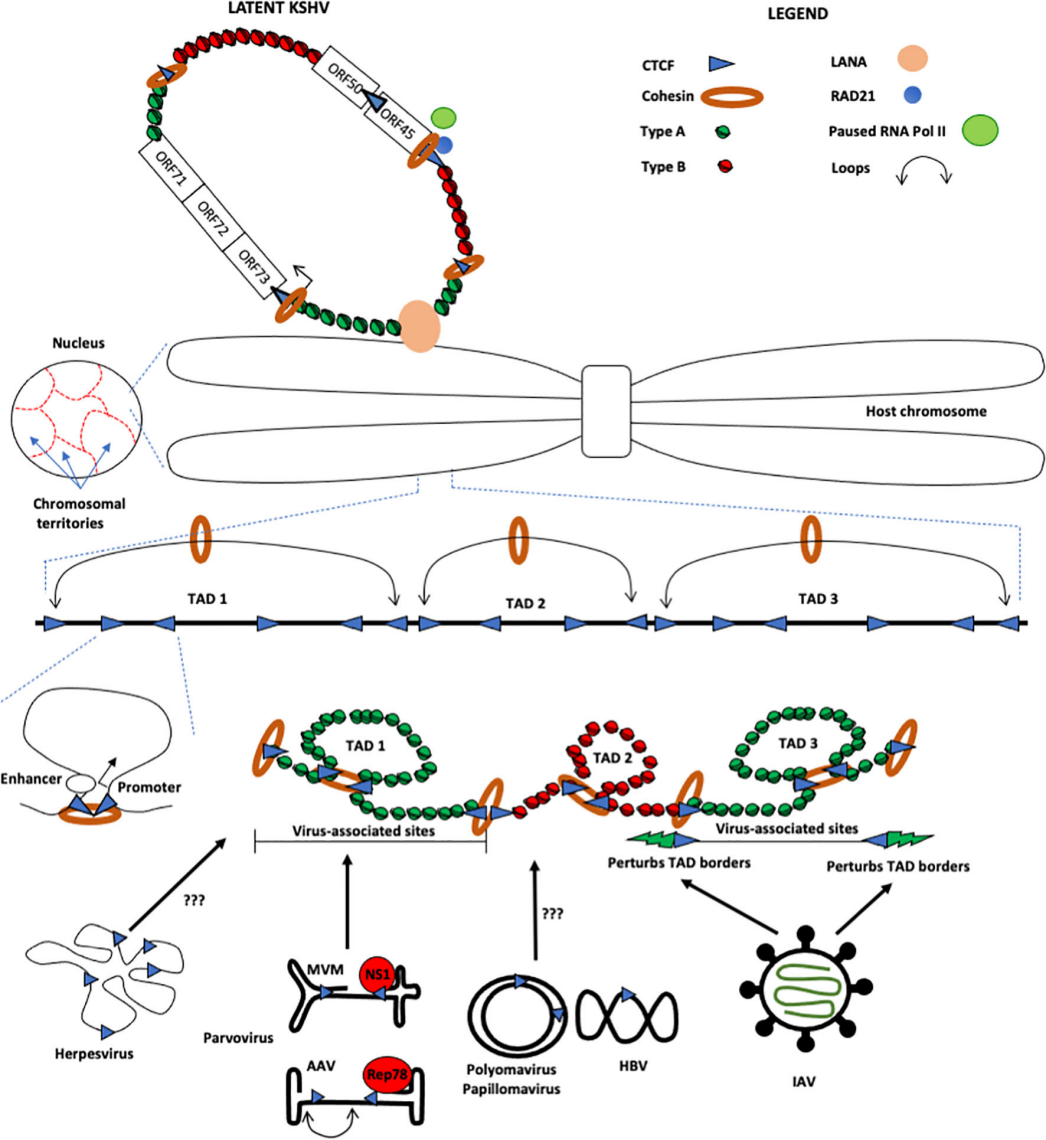
Schematic of how host chromosomes occupy nuclear territories and fold in-*cis* to form Topologically Associating Domains (TADs). These TADs are built up of multiple smaller *cis*-loops such as promoter-enhancer loops (as shown). These TADs are formed by interactions between convergent CTCF (blue triangle) and Cohesin (orange ring) bound regions, which modulate the TAD architecture and demarcate the boundaries between Type A-associated chromatin (green nucleosomes) from Type B-associated regions (red nucleosomes). Upon infection, Influenza A Virus (IAV) localizes to distinct TAD regions, which perturb the border between Type A and Type B chromatin (shown as green lightning), leading to eviction of cohesin from the borders and Type A chromatin into Type B. On the other hand, the protoparvovirus Minute Virus of Mice (MVM) localizes to distinct nuclear sites containing Topologically Associating Domains (TADs) that contain Type A chromatin (depicted in green histones). The borders between adjacent TADs are maintained by architectural proteins such as CTCF and Cohesin. However, in spite of the identification of CTCF binding sites on small DNA viruses (HBV, HPV, polyomaviruses etc) and herpesviruses, the mechanism of how they interact with host chromatin architecture remains to be elucidated. Also depicted (top, left) is Kaposi’s-sarcoma-associated herpesvirus (KSHV), which persists in latent form as chromatinized episomes that are tethered to host chromosomes by the latency associated nuclear antigen (LANA). CTCF, often along with Cohesin, bind the latent KSHV genome at many distinct sites (note that for simplicity, only a few are shown). CTCF/Cohesin are important boundaries between transcriptionally active (green nucleosomes) and silent (red nucleosomes) regions and are additionally important for coordinating physical interactions among the latency control region and the promoter regulatory region encoding the lytic immediate early protein RTA (ORF50; not shown).

The advent of chromosome conformation capture (3C) technologies has provided unprecedented insights into the mechanisms by which cellular DNA is spooled and packaged into the nuclear microenvironment, and how this packaging impacts biological processes (**Figure 1**). The original 3C methodology, described by Dekker and colleagues in 2002, enabled the detection of the frequency of contacts between pairs of genomic loci (1). Various iterations of this technology, reviewed elsewhere, were subsequently developed, providing essential tools to interrogate the principles of spatial organization of the genome (2, 3). Though these techniques have greatly improved our understanding of *cis*-folding processes of cellular DNA, these assays have also revealed that there exists little *trans*-interaction between host chromosomes (4). In spite of these features of mammalian genome looping, viral infection of host cells represents a common biological process where two genomes, host and viral pathogen, can interact with one another in-*trans*. Emerging studies have thus focused on unraveling how viral pathogens utilize the principles of genome folding to navigate the nuclear environment and establish infection. Here we briefly review some of the recent discoveries that have advanced our understanding of how DNA viruses co-opt host architectural proteins to organize their genomes, and in doing so, facilitate essential processes such as infection, lytic replication, and latency. In particular, we highlight the ability of a diverse array of DNA viruses to utilize genome-organizing protein CCCTC-Binding Factor (CTCF) in one or both of two ways: 1. to impact the chromatin topology of the host genome or 2. to organize its own genome in ways that enable sophisticated epigenetic control (**Figure 1**). Indeed, a wealth of evidence has established that human tumor viruses Kaposi’s sarcoma-associated herpesvirus (KSHV) and Epstein-Barr virus (EBV) persist as latent episomes in B lymphocytes, and that CTCF and/or Cohesin play important roles in the maintenance of these episomes (reviewed in 5). Episome maintenance is correlated with various virus-induced cancers, in part because latent episomes and latency gene products can impact host genome topology and, by extension, gene expression. Thus, a deeper understanding of the interactions among viral episomes, viral latency products, and host genomes will prove critical in our understanding of virus-induced oncogenesis as well as the design of targeted cancer therapeutics by engineered oncolytic viruses.

## TADs, Chromatin Loops as Dynamic Structures

At the simplest level, the mammalian genome is folded according to a distinct hierarchical order made up of promoter-enhancer loops [reviewed in (6)]. Many such functional loops, combined with structural loops, all generated within a distinct region of a chromosome spanning hundreds of kilobases, creates a topologically associating domain [TAD; **Figure 1**; (7)] Structurally defined by the high frequency of intradomain contacts over a large region of the chromosome, TADs are packaged in chromatin that has a similar transcriptional status. Functionally, this suggests that genes in the same TAD are coregulated (8, 9). This structural organization segregates the genome into chromatin types, broadly defined as Type A, which is largely associated with permissive chromatin, and Type B, which is composed of repressive chromatin [**Figure 1**; (4, 10)]. In addition to expression, the boundaries of TADs coincide with replication boundaries, suggesting that TADs can potentially replicate as stable units (8). Indeed, these units are established early in G1 phase of cell cycle and are dissolved pre-mitotically at G2 phase (11, 12). As the fidelity of the genetic code must be maintained during cell cycle, and since TAD structures oscillate between formation and dissolution throughout cell division, we can infer that TADs may play a role in maintaining the stability of the cellular genome (discussed below).

One of the most visible features of TAD boundaries is the binding of the architectural protein CTCF, which, when bound in a convergent orientation, enables the formation of a loop of the intervening DNA (4). By serving as a border between the high frequency intra-TAD interactions and low frequency inter-TAD interactions with neighbors, CTCF sites at TAD boundaries influence the maintenance of the local chromatin environment (13–15). The loop extrusion model, proposed to explain the mechanics of loop formation, posits that chromatin is extruded through the Cohesin complex until it encounters CTCF-bound distal elements, which form TAD boundaries (16). Convergence of the CTCF binding sites is critical for this process. The formation of a CTCF/Cohesin-dependent loop anchor sets up the milieu for the generation of subsequent smaller DNA loops in conjunction with Mediator, cohesin and Ying-Yang (YY1) proteins that constitute the entire TAD (6, 17, 18). These loop anchors at TAD boundaries, bound by CTCF and Cohesin, are vulnerable to DNA damage in a transcription-, replication-, and cell-type-independent, but Cohesin-dependent manner until they encounter convergent CTCF-bound distal elements (19, 20). Additionally, the topoisomerase subunit protein Top2b regulates the torsional stress at these loop anchors to modulate genome stability (20). Taken together, replication stress initiated at these sites during genome replication predisposes them to becoming fragile genomic regions, which can serve as the initiation sites for chromosomal rearrangements and genome instability (21).

## Utilization of Genome Folding Principles by Viral Pathogens

DNA viruses enter cells through receptor-mediated endocytosis, traverse the cytoplasm, and enter the nucleus through nucleopore dependent or independent mechanisms, where they usurp cellular factors to replicate their genomes and generate progeny virions (22, 23). Upon infection of host cells, viruses can induce or inhibit a cellular DNA damage response (DDR), potentially downstream of innate immune signals, caused by the presence of foreign DNA or viral proteins (24). Modulating the host DDR has the potential to impede [adenovirus and herpesviruses (25–30)] or facilitate [DNA tumor viruses and parvoviruses (31–35)] virus infection. As viral pathogens continue to replicate in the host nucleus, the viral nucleic acids and proteins or the DDR they modulate, have the capacity to modulate the topology of host chromatin to benefit viral infection. This has recently been observed in for Influenza A Virus (IAV), which in spite of being an RNA virus, replicates in the nucleus using the IAV Non-Structural Protein (IAV-NS1), which inhibits transcription termination at the ends of highly transcribed genes. This leads to the displacement of Cohesin from CTCF sites (36), converting repressive chromatin compartments (Type B; (4) into permissive ones [mostly Type A; [**Figure 1**; (4)]. These changes in the epigenetic landscape of infected cells by readthrough-transcription modify the host’s TADs, especially at the borders maintained by CTCF and Cohesin. Interestingly, changes in TAD structure by readthrough-transcription of the host genome have also been observed during infection of the unrelated DNA virus Herpes Simplex Virus [HSV (37)], indicating that targeting the host genome’s topology may be an efficient mechanism for viral pathogens to gain a foothold in the nuclear environment. By modulating cellular TADs, viral pathogens may increase the chromatin environment that is available to them. They can then use this environment to establish and expand their replication centers while simultaneously evading host antiviral defense factors.

In addition to its impact on the host genome’s topology, viruses must also utilize cellular factors to appropriately express proteins essential for their life cycle. In this regard, the large 150 kb genome of HSV forms multiple DNA loops, not unlike TADs in the host cells, which are maintained by CTCF-bound elements and are essential for viral infection, latency and reactivation (38, 39). Additionally, a wealth of evidence now indicates that human gammaherpesviruses, known for their biphasic life-cycles involving latency and lytic replication, utilize CTCF and Cohesin to establish and maintain latency, and thus, life-long infection. Recent work has established that CTCF and/or Cohesin contribute to viral latency by acting as boundary factors and, in some cases, coordinating 3D genome looping; these events enable viral genomes to adopt a heterochromatin structure that limits viral gene expression to a few latent genes (40). This manipulation of viral 3D genomic architecture is linked to the ability of herpesviruses to elicit cellular transformation and oncogenesis (41, 42).

## Human Gammaherpesviruses Manipulate Higher-Order Genomic Architecture to Establish Latency and Promote Oncogenesis in B cells

The human gammaherpesviruses, Kaposi’s-sarcoma-associated herpesvirus (KSHV) and Epstein-Barr Virus (EBV), can establish latency in B lymphocytes, allowing both viruses to simultaneously avoid immune surveillance while creating a long-term infective niche. Indeed, both KSHV and EBV have evolved sophisticated mechanisms to preserve latent genome maintenance in proliferating cells; this is thought to be a major contributing factor in the onset of oncogenesis (42, 43). Both KSHV and EBV establish latency through a wide array of epigenetic mechanisms that have been reviewed elsewhere (40, 44). Briefly, however, these mechanisms include regulation by non-coding RNAs (ncRNAs), post-translational modification (PTM) of histones, and DNA methylation. As each of these events has the potential to disrupt TAD boundaries, all are potential drivers of tumorigenesis (40, 45). Recent work, however, has focused on the mechanisms by which these viruses manipulate higher-order chromatin architecture in order to establish and preserve latency, as well as the impact of latent virus-induced epigenetic modifications on the host genome (40, 42, 44).

### CTCF and Cohesin Regulate Latency in KSHV-Infected Cells

KSHV has a linear double-stranded DNA genome of approximately 160-170 kb. Upon cellular entry, KSHV genomes are circularized into chromatinized episomes that persist in the nucleus of infected cells. Though extrachromosomal, the viral episomes are tethered to the host chromosome by the latency associated nuclear antigen (LANA), which bridges viral DNA and host chromatin, including histones H1, H2A, and H2B (46–50). Since the extrachromosomal viral genomes are associated with host histones and are, by extension, substrates for chromatin modifiers, it was reasonable to hypothesize that eukaryotic chromatin organizing factors such as CTCF and Cohesin may bind to KSHV episomes. Indeed, a CTCF site was found within the latency control region; specifically, the first intron of the multicistronic transcript encoding key latency genes LANA (ORF73), vCyclin (ORF72), and vFLIP [ORF71; (**Figure 1**)]. This site colocalized with a Cohesin binding site and notably, disruption of the viral CTCF binding site abrogated Cohesin binding (51). A subsequent report defined, using ChIP-Seq, the presence of at least 25 additional CTCF binding sites throughout the KSHV genome, many of which colocalized with Cohesin (52). Viral genome-wide 3C methods established that CTCF-Cohesin interactions are critical for organizing KSHV genomes into chromatin loops (53). Indeed, CTCF and Cohesin coordinate physical interactions between the KSHV latency control region and the promoter regulatory region encoding the lytic immediate early protein RTA (ORF50), as well as between the 5’ and 3’ ends of the latency transcription cluster, both of which contribute to the control of KSHV latency. In agreement with these findings, mutation of CTCF-Cohesin binding sites or depletion of Cohesin subunits leads to a rapid reactivation of lytic gene expression, suggesting that the CTCF-Cohesin binding at the latency control region is critical for repressing the transcription of lytic genes (52–54). Though CTCF and Cohesin are clearly important for the maintenance of KSHV latency, both are, somewhat paradoxically, positioned upstream of the divergent promoter of the immediate early genes encoding RTA (ORF50) and ORF45, both key factors in the lytic cycle. Notably, Cohesin subunit Rad21 was shown to be important for retaining RNA Polymerase II at the promoter of ORF45, where it is poised to respond to reactivating signals that will drive the transcriptional up-regulation of various lytic genes [**Figure 1**; (54)].

Though numerous studies have established that KSHV is inextricably linked to the etiology of the B cell malignancies primary effusion lymphoma [PEL (55)] and multicentric Castleman’s disease [MCD (56)], the precise mechanisms by which KSHV latency contributes to tumorigenesis are not yet clear. Many of KSHV’s latent gene products have been shown to influence the host epigenome. Given its close association with chromatin, it is hardly surprising that LANA impacts host gene expression; in many instances, this is due to alterations in DNA methylation patterns or histone modifications (42). LANA also cooperates with latency factor vFLIP in the transcriptional up-regulation of methyltransferase EZH2 (57). Additionally, infection with a mutant virus that does not express KSHV-encoded miRNAs resulted in a near-complete disruption of DNA methylation within the viral and host cell genomes (42, 58). Thus, there is ample evidence that KSHV latency products alter both the host epigenetic landscape and host gene expression, both of which may contribute to the onset of oncogenesis. It should be noted, however, that KSHV latency products are insufficient to drive tumorigenesis in mouse models (42). Thus, it is possible that latency products may cooperate with transiently-expressed lytic gene products in inducing epigenetic changes in the host genome that drive tumorigenesis. Indeed, there is a growing body of evidence that in some cellular contexts, certain lytic genes can be expressed during latency (42, 59). Given that CTCF and Cohesin are key players in regulating the switch between latent and lytic gene expression, they may play a role in driving less “rigid” latency gene expression programs that contribute to cellular transformation.

### CTCF Regulates Differential Latency Types in EBV-Infected B Cells

The other human gammaherpesvirus, Epstein Barr Virus (EBV, or HHV4), can establish life-long infection in more than 90% of the population worldwide. Its highly effective pathogenesis can be attributed, in part, to its ability to manipulate the host cell’s nuclear environment to its advantage, establishing a variety of transcriptional programs that allow it to adapt to changing environmental conditions (41). Though this is the result of a complex set of epigenetic changes, a large body of work has established that CTCF is an active participant in the higher order spatial organization of the EBV genome, thus contributing to the regulation of latency and in turn, driving oncogenesis. Much like KSHV, EBV has a linear, double-stranded DNA genome that, upon entry into the host nucleus, becomes circularized as a result of recombination events involving terminal repeats present at each end of its genome (60, 61). As is the case with the host genome, the latent EBV episome folds into transcriptionally active and repressed regions, which are separated into topologically distinct loops (43). In contrast to KSHV latency, where a relatively fixed subset of genes are expressed, EBV gene expression during latency correlates with differential utilization of promoter elements and transcription start sites; these distinct gene expression programs are referred to as latency types (62, 63). Type III latency, which is the least restrictive, involves expression of the EBNA (-1, -2, -3A, -3B, -3C and -LP) proteins, non-coding RNAs, and latency membrane proteins LMP-1 and -2 (64). In Type I latency, by contrast, gene expression is restricted to EBNA-1 and several non-coding RNAs (65, 66). The epigenetic landscape differs substantially between latency types, though the histone modifications correlate with expected marks at regions containing transcriptionally active (Type A) or repressive (Type B) chromatin. In many cases, these domains have distinct boundaries marked by CTCF binding sites. Indeed, CTCF binds the EBV genome at a minimum of 19 distinct sites, most at key promoters that regulate latency-associated genes (67–69). In the case of the Qp promoter, which regulates EBNA-1 transcription, CTCF binds adjacent to a region enriched for DNA methylation and the repressive H3K9me3 mark. If CTCF binding is abrogated, these repressive chromatin marks spread into the Qp promoter start site, silencing transcription (67). This finding underscores a role for CTCF as a boundary factor that can, in effect, enforce distinct latency programs. Additional evidence indicates that CTCF binds between the Cp promoter, which drives expression of EBNA-LP, EBNA-2, EBNA-3A, EBNA-3C, and EBNA-1, and OriP, the distal enhancer for this promoter. Thus, CTCF may act as an insulator that regulates Cp promoter activity in Type I latency (70).

In addition to its purported roles as an insulator and a boundary factor, several studies have also suggested that CTCF promotes long-range interactions between promoter and enhancer regions (41). Indeed, 3C analysis of the EBV genome identified the formation of chromatin loops between the enhancer region OriP and, depending on latency type, either the Cp (Type I) or Qp (Type III) promoter (71). Mutation of CTCF binding sites adjacent to the Cp and Cq promoters disrupted loops bringing each respective promoter into proximity to the OriP enhancer, indicating that CTCF binding orchestrates the formation of distinct chromatin loops that drive EBV latency type. Thus, these CTCF-mediated changes in chromatin architecture drive differential promoter targeting by the OriP enhancer and consequently, expression of distinct latency gene products (71). A wealth of evidence, reviewed elsewhere, indicates that EBV latency gene products influence host genome topology (41). For example, EBNA-1 contributes to telomere dysfunction during latent EBV infection (72, 73), and since it can bind to a variety of host genomic sites, may alter chromatin structure or nucleosome positioning at those sites (74–78). Notably, a recent study indicated that EBNA-3C modulates the conformation of the B cell epigenome by interfering with the looping among the genes for tumor suppressors *p14^ARF^*, *p16^INK4A^*, and *p15^INK4B^* at the CDKN2A/2B loci. Thus, EBNA-3C disrupts physical interactions among the promoters of the three genes, suppressing their transcription and by extension, interfering with their expression (79). Additionally, changes in the 3D architecture of the B cell epigenome by EBV oncoproteins likely regulate B cell transformation by altering MYC expression (79). This work provides important insight into how EBV-driven genome organization can drive cellular transformation.

## Manipulation of 3D Genomic Architecture by Small DNA Viruses

With regard to small DNA viruses, it is tempting to speculate that they also adopt a looped conformation akin to cellular promoter-enhancer loops. Interestingly, the genome of the *Dependoparvovirus* Adeno-Associated Virus 2 (AAV2), which is 5 kilobases long, has been observed to adopt a looped structure in electron microscopy studies [**Figure 1**; (80)]. Additionally, Cohesin subunits SMC5/6 interact with the HBx protein of Hepatitis B Virus (HBV) during infection to block extrachromosomal DNA transcription (81). If the degradation of SMC5/6 complex is required for HBV expression in host cells, then this would also impact the host genome’s topology. Alternatively, HBx-mediated degradation of SMC5/6 may be targeted to the subnuclear locations in proximity to the HBV genome. However, further studies on the *cis*-topology of small DNA viruses are yet to be investigated using chromosome conformation capture techniques.

The genome of the carcinogenic Human Papillomavirus 18 (HPV18) adopts a looped configuration maintained by interaction between distally bound CTCF and YY1, which inhibits the expression of the HPV oncogenes E6 and E7 in undifferentiated cells (82). The HPV oncogene E2 interacts with SMC5/6 to facilitate genome maintenance, which may involve maintaining viral genome looping (83). Strikingly, mutation of the CTCF binding site on the HPV genome leads to alterations in processing of viral RNA transcripts (84). These findings are consistent with newly identified roles of CTCF in RNA processing for both the host and small DNA viruses such as Parvoviruses (85–87). In order to investigate where the genome of the non-integrating Parvovirus Minute Virus of Mice (MVM) localizes, Majumder et al., 2018 utilized a modified form of 4C-seq assay to investigate the localization of small DNA viruses to cellular sites, a technique that has been dubbed V3C-seq [Viral Chromosome Conformation Capture Assays; (35)]. These studies revealed that MVM, which requires cellular DDR to replicate, also localizes to cellular sites of DNA damage, many of which are previously identified Early Replicating Fragile sites [ERFs (21)]. Strikingly, many of these sites also coincide with TADs and contact domains that are packaged in Type A chromatin (35). In subsequent studies, Majumder and colleagues discovered that ectopically expressed viral non-structural phosphoprotein NS1 binds to cellular sites of DNA damage, and, when bound cognate sequences on the viral genome, can transport the viral genome to these cellular DDR sites [**Figure 1**; (88)]. These findings suggest that the NS1 protein of MVM, a small DNA virus, can help a viral genome navigate the nuclear milieu to essential TAD/DDR regions that promote viral infection.

## Discussion

In the years since the first report of chromosome conformation capture technology (1) we have gained unprecedented insight into the mechanisms of genome folding and how this regulates cellular physiology. A wealth of recent work has focused on unraveling how viral pathogens utilize the principles of genome folding to navigate the nuclear environment and establish infection. Future studies are likely to shed light on additional mechanisms at work between viral and host genomes. In this regard, Chromatin Interaction Analysis with Paired-End Tag Sequencing [ChIA-PET; (89)] assays of viral proteins, which combine Chromatin Immunoprecipitation with high-throughput Chromosome Conformation Capture (3C) techniques, will yield critical insights into how both viral and host proteins modulate nuclear topology, establish viral replication centers, affect latency and impact reactivation as a part of viral pathogenesis. Thus far, these studies have only been performed for RNA Polymerase II in EBV-infected B cells to characterize the EBV regulome in lymphoblastoid cells, identifying critical insights into how spatial organization underlies EBV-dependent cellular transformation (79). Additionally, as viral infection can activate or inhibit DNA damage signaling [reviewed in (22)], and the cellular DDR induces alterations in cellular chromosome conformation (90), the cause-effect relationship between viral infection, DNA damage and chromatin conformation are yet to be unraveled. These mechanisms may be unraveled by ChIA-PET studies of bridging molecules such as CTCF, Cohesin and gamma H2AX between the virus and host genomes, which will yield critical insights into the mechanisms of viral latency, reactivation, virus-induced oncogenesis and how viral oncolytic agents may function.

## Author Contributions

KM and AM both made substantial and direct contributions to the writing and editing of the manuscript and have approved it for publication. All authors contributed to the article and approved the submitted version.

## Funding

KM is supported by a NIH K99/R00 Pathway to Independence Award (AI148511), University of Wisconsin Office of Vice Chancellor for Research and Graduate Education and the University of Wisconsin Carbonne Cancer Center’s Human Virology Program. Additional support for this work was provided by a PSC-CUNY Award, jointly funded by The Professional Staff Congress and the City University of New York (TRADA-51-497; AM).

## Conflict of Interest

The authors declare that the research was conducted in the absence of any commercial or financial relationships that could be construed as a potential conflict of interest.
